# Supplementary material for: Combined NMR Spectroscopy and Quantum-Chemical Calculations in Fluorescent 1,2,3-Triazole-4-carboxylic Acids Fine Structures Analysis
Source: Int J Mol Sci. 2023 May 18;24(10):8947. doi: 10.3390/ijms24108947 (PMC10219572; doi:10.3390/ijms24108947)
Supplement: Supplementary file 1 [file ijms-24-08947-s001.zip › ijms-2386428-supplementary.pdf]

Combined NMR spectroscopy and quantum-chemical calculations in Fluorescent 1,2,3-triazole-4-carboxylic acids fine structures analysis

Nikita E. Safronov[a], Irena P. Kostova[b], M. Alcolea Palafox[c**], Nataliya P. Belskaya[a*]

[a]Ural Federal University, 19 Mira Str., Yekaterinburg 620002, Russian Federation
[b]Department of Chemistry, Faculty of Pharmacy, Medical University – Sofia, 2 Dunav Str., Sofia, Bulgaria
[c]Departamento de Química Física, Facultad de Ciencias Químicas, Universidad Complutense, Madrid 28040, Spain

Corresponding authors Email address: n.p.belskaya@urfu.ru (Nataliya P. Belskaya)

Table of contents

1. Experimental section S2
2. NMR spectra of new compounds S5
3. The experimental determination of the pKa values of acids 1a and 1b S9
4. Photophysical investigation S11
5. NMR spectra of acids 1a and 1b compounds in different solvents S15
6. Reference S25

1. Experimental Section

Instruments and Materials

^{1}H NMR and ^{13}C NMR, spectra were recorded with a Bruker Avance II (Karlsruhe, Germany) (400 MHz for ^{1}H, 100 MHz for ^{13}C) spectrometer. Chemical shifts are reported in parts per million (ppm) relative to TMS in ^{1}H NMR, to the residual solvent signals in ^{13}C as external reference. Coupling constants (*J*) values are given in Hertz (Hz). Signal splitting patterns are described as a singlet (s), doublet (d), triplet (t), quartet (q), sextet (sext), quintet (quin), multiplet (m), broad (br), doublet of doublets (dd), doublet of triplets (dt) or AA′XX′ - spin system of para-substituted benzene with two different substituents. Mass spectra were recorded with a Shimadzu GCMS-QP 2010 "Ultra" (Kyoto, Japan) mass spectrometer using the electron impact (EI) ionization technique (40-200 °C, 70 eV). The abbreviation $[M]^+$ refers to the molecular ion. Spectra of exact mass were acquiring on a quadrupole orthogonal acceleration time-of-flight mass spectrometer (Synapt G2 HDMS, Waters, Milford, MA). Samples were infused at 3uL/min and spectra were obtained in positive (or: negative) ionization mode with a resolution of 15000 (FWHM) using leucine enkephalin as lock mass. The Fourier-transform infrared (FT-IR) spectra were obtained using a Bruker Alpha (NPVO, ZnSe) spectrometer (Ettlingen, Germany). Melting points were determined on a Stuart SMP3 apparatus (Staffordshire, ST15 OSA, UK).

UV-Vis absorption spectra were recorded on a Shimadzu UV-1800 spectrometer. The fluorescence of the sample solution was measured using a Hitachi F-7000 spectrophotometer (Tokyo, Japan). The absorption and emission spectra were recorded in DMSO, 1,4-dioxane, MeOH using 10.00 mm quartz cells. The excitation wavelength was at the absorption maxima. Atmospheric oxygen contained in solutions was not removed. Concentration of the compounds in the solution was 5.0×10^{-5} M and 5.0×10^{-6} M for absorption and fluorescence measurements, respectively. The relative fluorescence quantum yields (QY) were determined using quinine sulfate in 0.1 M H_2SO_4 as a standard ($\Phi_F = 0.546$). Time-resolution study were recorded on Horiba FlouroMax 4 Spectrofluorometer (Kyoto, Japan) with Quanta-φ integrating sphere using FluorEssence 3.5 Software.

The reactions were monitored by analytical thin-layer chromatography (TLC) on aluminium-backed silica-gel plates (Sorbfil UV–254). Visualization of components was accomplished by short wavelength UV light (254 nm). Solvents were dried and distilled according to the common procedures. All solvents were of spectroscopic grade.

1-aryl-4-cyano-1,2,3-triazoles **3a-r** were prepared according to procedures reported in the literature and their spectral characteristics were identical to the published data [1,2].

Preparation and characterization of new compounds

General synthetic procedure for synthesis acids 1

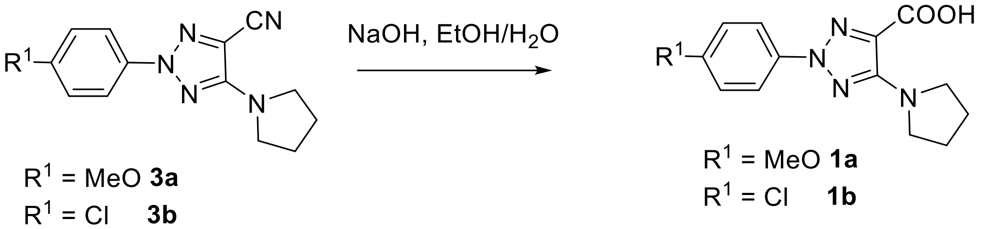

R^1 = MeO **3a**
R^1 = Cl **3b**

R^1 = MeO **1a**
R^1 = Cl **1b**

Triazole **3a, b** (1.0 mmol) was added to a 40% NaOH solution in ethanol/H_2O. The mixture was heated under reflux until TLC indicated total conversion of starting triazole. Then solution was cooled to room temperature and poured into a mixture of water and crushed ice. The resulting mixture was acidified with conc. HCl to pH 1 and formed precipitate was filtered off and crystallized from ethanol.

General synthetic procedure for synthesis salts 2

Aqueous sodium hydroxide solution (1.0 mmol, 2 mL) was added dropwise to a solution of 5-amino-2-aryl-1,2,3-triazol-4-carboxylic acid (1.0 mmol) in ethanol (10 mL) at 60 °C. The mixture was stirred at 60 °C for a further 30 - 40 min. Volatile solvents were removed in vacuo to dryness to provide sodium 5-amino-2-aryl-1,2,3-triazol-4-carboxylate as a solid.

2-(4-Methoxyphenyl)-5-(pyrrolidin-1-yl)-2*H*-1,2,3-triazole-4-carboxylic acid (**1a**). Colorless powder,

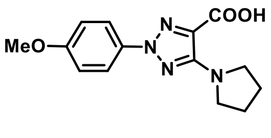

yield 93%, mp 184-185 ºC. ^{1}H NMR (DMSO-d_6, 400 MHz): δ 1.88 (m, 4H, CH_2), 3.44 (t, 4H, J = 6.3 Hz, CH_2), 3.81 (s, 3H, OMe), 7.08 and 7.84 (AA′XX′, 4H, J = 8.9 Hz, H_{Ar}), 12.89 (s, 1H, COOH). ^{13}C NMR (DMSO-d_6, 100 MHz.): 24.9 (2C), 49.8 (2C), 55.5, 114.6 (2C), 119.6 (2C), 125.6, 132.5, 154.8, 158.5, 162.2. HRMS (ESI+), *m/z* calcd. for $C_{14}H_{16}N_4O_3$ $[M+H]^+$ 289.1295, found 289.1299.

2-(4-Chlorophenyl)-5-(pyrrolidin-1-yl)-2*H*-1,2,3-triazole-4-carboxylic acid (1b). Colourless powder,

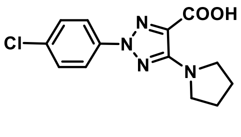

yield 89%, mp 132-133 °C. ^{1}H NMR (DMSO-d_6, 400 MHz): δ 1.89 (m, 4H, CH_2), 3.44 (m, 4H, CH_2), 7.58 and 7.92 (AA′XX′, 4H, J = 8.8 Hz, H_{Ar}), 13.07 (s, 1H, COOH). ^{13}C NMR (DMSO-d_6, 100 MHz.): 24.9 (2C), 49.8 (2C), 119.5 (2C), 126.8, 129.5 (2C), 131.5, 137.5, 154.8, 162.0. HRMS (ESI+), *m/z* calcd. for $C_{13}H_{13}ClN_4O_2$ $[M+H]^+$ 293.0800, found 293.0809.

sodium 2-(4-methoxyphenyl)-5-(pyrrolidin-1-yl)-2H-1,2,3-triazole-4-carboxylate (**2a**).

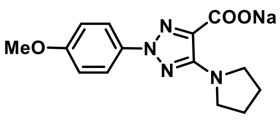

Colourless powder, yield 80%, mp > 350 ºC. ^{1}H NMR (D_2O, 400 MHz): δ 1.95 (s, 4H, CH_2), 3.38 (s, 4H, CH_2), 3.85 (s, 3H, CH_3), 7.05 (d, 2H, J = 8.4 Hz, H_{Ar}), 7.40 (d, 2H, J = 8.4 Hz, H_{Ar}). ^{13}C NMR (D_2O, 100 MHz.): 24.8, 50.4 (2C), 55.6 (2C), 114.6 (2C), 120.5 (2C), 132.4, 133.0, 154.1, 158.0, 168.7. HRMS (ESI+), *m/z* calcd. for $C_{14}H_{15}N_4NaO_3$ $[M+H]^+$ 289.1295, found 289.1306.

Sodium 2-(4-chlorophenyl)-5-(pyrrolidin-1-yl)-2H-1,2,3-triazole-4-carboxylate (**2b**).

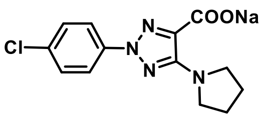

Colourless powder, yield 75%, mp > 350 ºC. ^{1}H NMR (D_2O, 400 MHz): δ 1.95 (s, 4H, CH_2), 3.38 (s, 4H, CH_2), 3.85 (s, 3H, CH_3), 7.41 (d, 2H, J = 7.4 Hz, H_{Ar}), 7.69 (d, 2H, J = 7.4 Hz, H_{Ar}). ^{13}C NMR (D_2O, 100 MHz.): 24.9 (2C), 50.3 (2C), 119.7 (2C), 128.9 (2C), 131.8, 133.6, 137.4, 154.3, 168.6. HRMS (ESI+), *m/z* calcd. for $C_{13}H_{12}ClN_4NaO_2$ $[M+H]^+$ 293.0800, found 293.0807.

2. NMR spectra of new compounds

1a

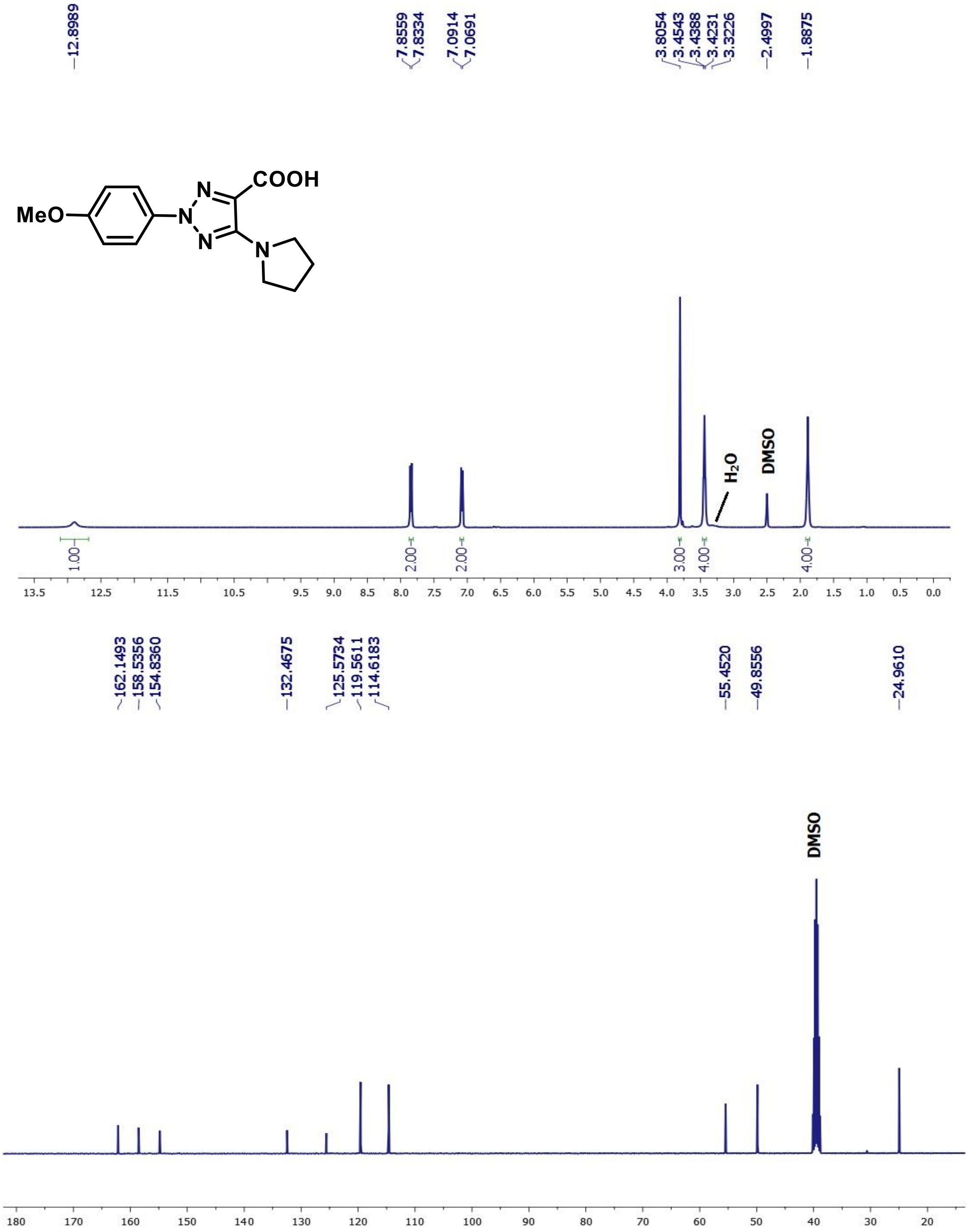

Fig. S1. ^{1}H NMR (400 MHz, DMSO- d_6) and ^{13}C NMR (100 MHz, DMSO-d_6) spectra of 2-(4-methoxyphenyl)-5-(pyrrolidin-1-yl)-2*H*-1,2,3-triazole-4-carboxylic acid (**1a**)

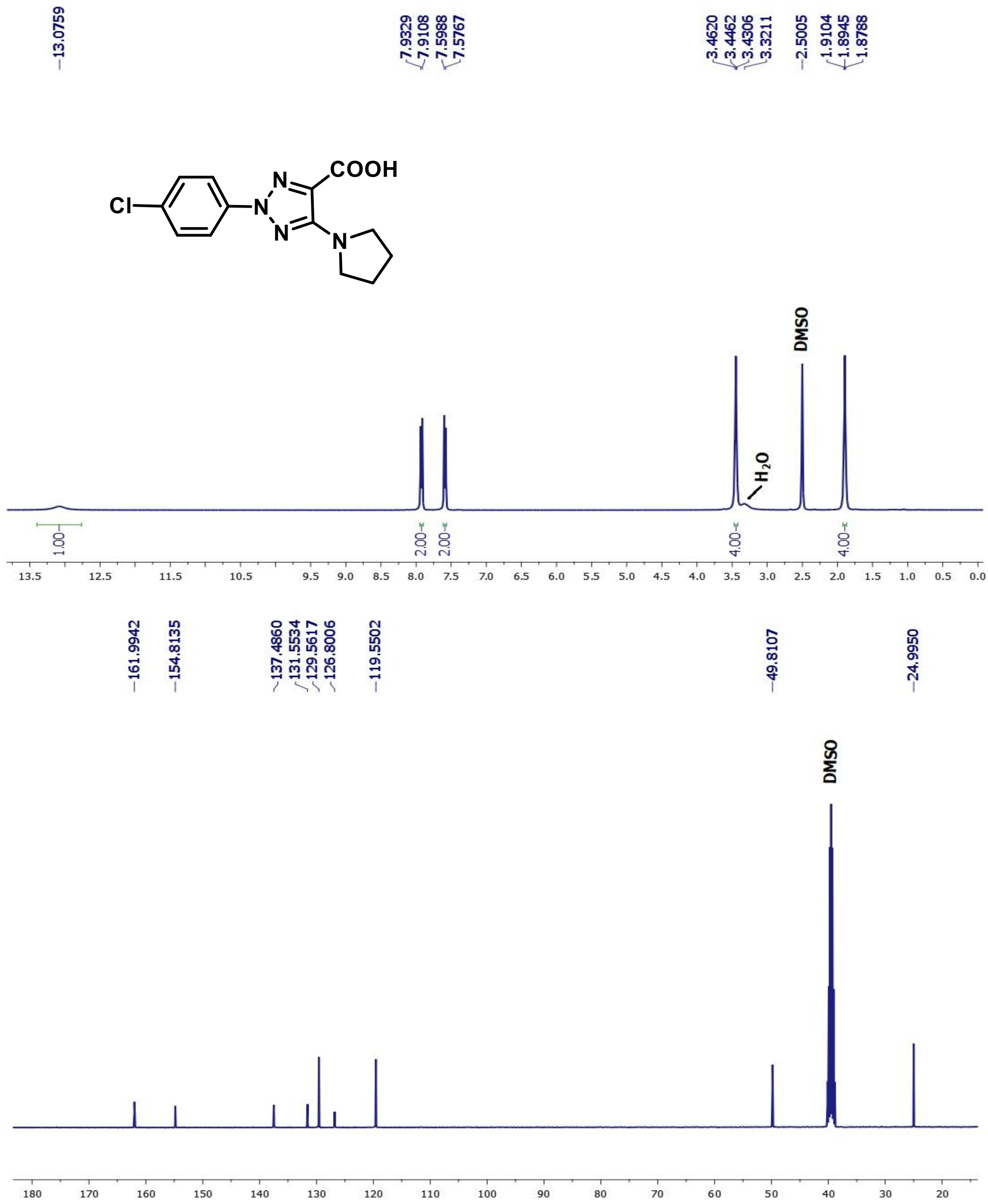

Fig. S2. ^{1}H NMR (400 MHz, DMSO- d_6) and ^{13}C NMR (100 MHz, DMSO-d_6) spectra of 2-(4-chlorophenyl)-5-(pyrrolidin-1-yl)-2*H*-1,2,3-triazole-4-carboxylic acid (**1b**)

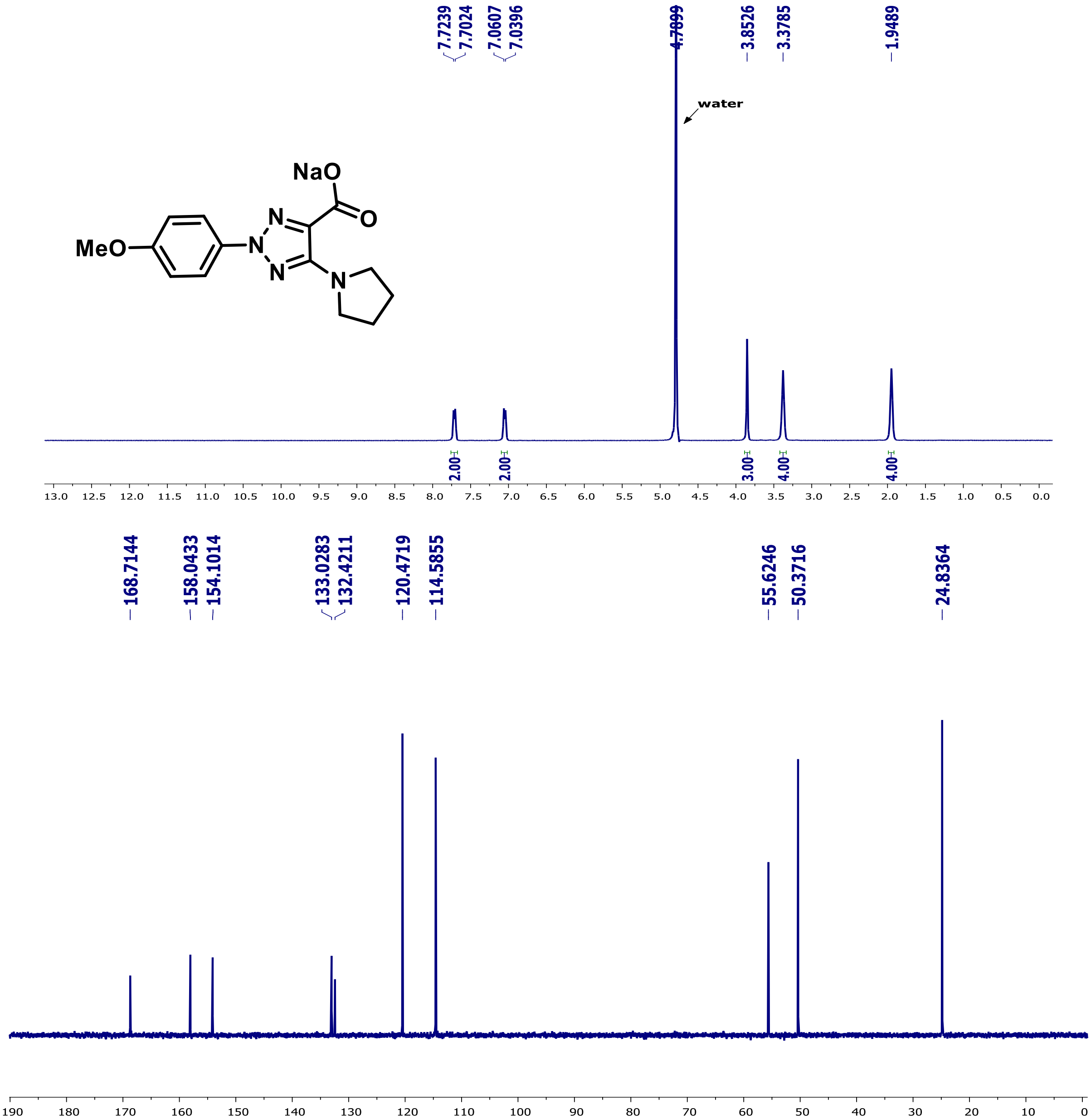

Fig. S3. ^{1}H NMR (400 MHz, D_2O) and ^{13}C NMR (100 MHz, D_2O) spectra of sodium 2-(4-methoxyphenyl)-5-(pyrrolidin-1-yl)-2H-1,2,3-triazole-4-carboxylate (**2a**).

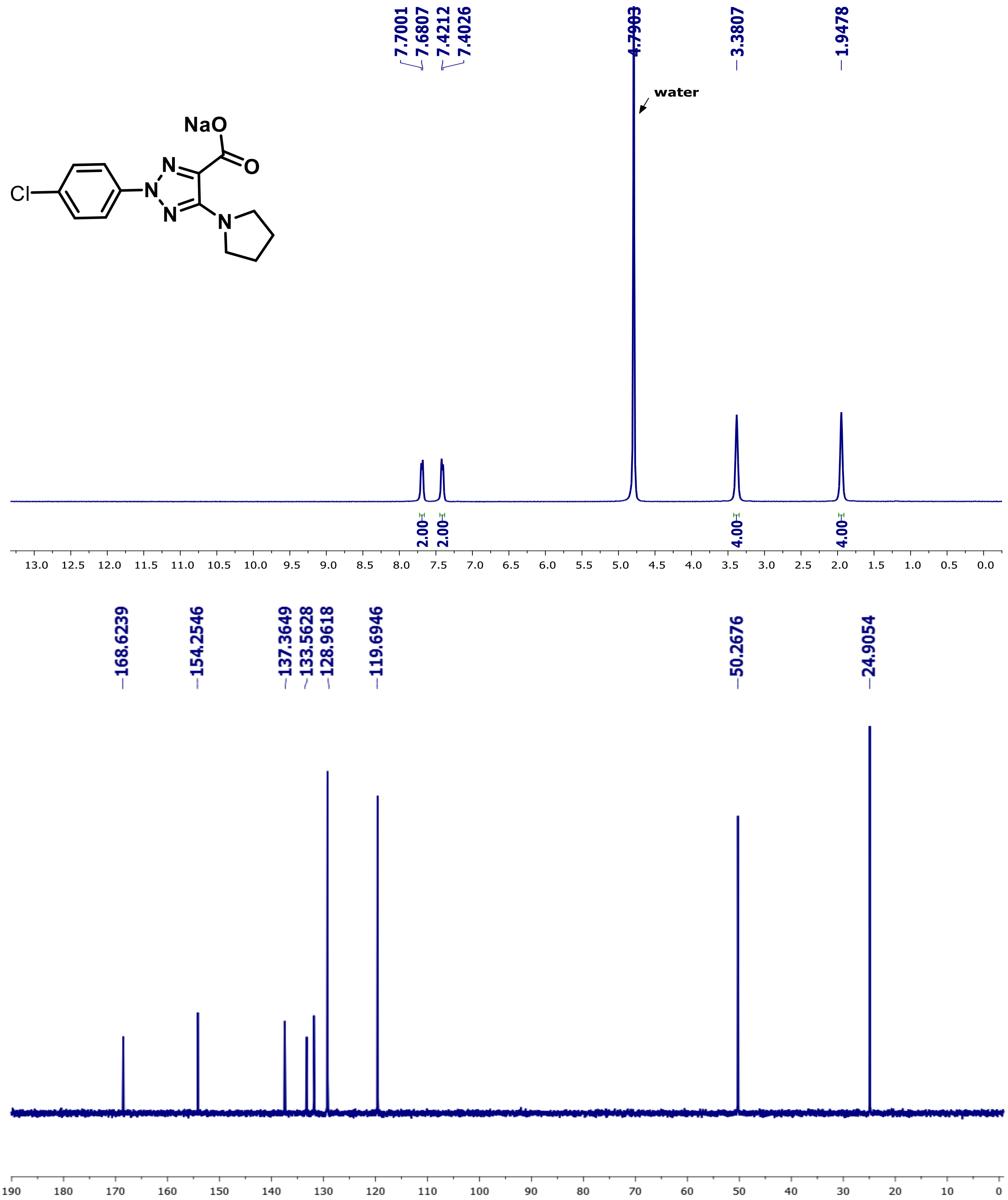

Fig. S4. ^{1}H NMR (400 MHz, D_2O) and ^{13}C NMR (100 MHz, D_2O) spectra of sodium 2-(4-chlorophenyl)-5-(pyrrolidin-1-yl)-2H-1,2,3-triazole-4-carboxylate (**2b**).

3. The experimental determination of the pKa values of acids 1a and 1b

The calculation was based on the ratio of the solution's concentration and pH [3].

This is possible because of the relationship between pKa and pH:

$$pH = pK_a + \lg ([A^-] / [AH]) \quad (1)$$

Where the square brackets are used to indicate the concentrations of the acid and its conjugate base. The equation may be rewritten as:

$$K_a/[H^+] = [A^-] / [AH]$$

This shows that pKa and pH are equal when half of the acid has dissociated. The buffering capacity of a species or its ability to maintain pH of a solution is highest when the pKa and pH values are close. So, when selecting a buffer, the best choice is the one that has a pKa value close to the target pH of the chemical solution. At half-neutralization the ratio $[A^-]/[HA] = 1$; since log(1) = 0, the pH at half-neutralization is numerically equal to pK_a. Conversely, when pH = pK_a, the concentration of HA is equal to the concentration of A^-. If the pH is known, the ratio may be calculated. This ratio is independent of the analytical concentration of the acid.

A 5×10^{-5} M DMSO-water (1:9, v/v) solutions of ATAs **1a, b** are found to have an average pH of 6.38 and 5.81 respectively. The concentration of hydrogen ions can be calculated with the formula $[H^+] = 1/10^{pH}$ M. If the concentration of hydrogen ions $[H^+] = 1/10^{pH}$ = x M, then:

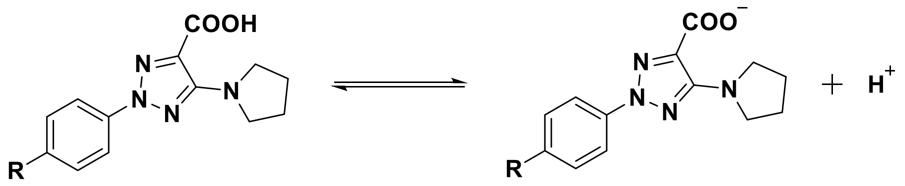

R = MeO (1a), Cl (1b)

Initial conc:	$5\cdot10^{-5}$ M		
changes:	-x M	+x M	+x M
equil conc:	$(5\cdot10^{-5} – x)$ M	x M	x M

The dissociation constant can be calculated with the formula $Ka = [H^+]\cdot[A^-]/[HA]$. With the known value of x: $Ka = x\cdot x /(5\cdot10^{-5} – x) = [H^+]^2/([HA]-[H^+])$.

pH values of **1a:** 6.41, 6.32, 6.40.

pH values of **1b:** 5.78, 5.80, 5.84.

pKa calculation for **1a**:

$^{1a}pKa_I$:

$[H^+] = 1/10^{pH} = 1/10^{6.41} = 3.89 \cdot 10^{-7}$ M

$Ka = (3.89 \cdot 10^{-7})^2$ M/ $(5 \cdot 10^{-5} - 3.89 \cdot 10^{-7}) = 3.05 \cdot 10^{-9}$

$^{1a}pKa_I = -\log(Ka) = -\log(3.05 \cdot 10^{-9}) = 8.52$

pKa values of **1a, 1b** are calculated with the same method.

Standard deviation and relative standard deviation calculations are also the same for all pKa values of **1a,b**

$$S_{Dev} = \sqrt{\frac{\sum(pKa - pKa^{avg})^2}{n-1}}$$

$$S_{Dev}^{r} = \frac{100S}{pKa^{avg}}$$

Confidential interval Θ for pKa values of **1a, b** can be found with Student's t-distribution:

$$\Delta pKa = \pm \frac{St_{p,f}}{\sqrt{n}}, f = n - 1, p = 0.95$$

Table S1. Results for the determination of pKa acids **1a** and **1b**

Compd	pH	pKa	Avg. pKa	S_{Dev}	S_{Dev}^{r}	Δ pKa	CI (Θ)
**1a**	6.41	8.52	8.5	0.047	0.554	±0.1	8.5±0.1
	6.37	8.43					
	6.40	8.50					
**1b**	5.78	7.24	7.3	0.061	0.837	±0.2	7.3±0.2
	5.80	7.28					
	5.84	7.36					

4. Photophysical investigations.

UV-Vis absorption spectra were recorded on a Shimadzu UV-1800 spectrometer. Fluorescence of the sample solutions was measured using a Hitachi F-7000 spectrophotometer (Tokyo, Japan). The absorption and emission spectra were recorded in Toluene, Dioxane, CH_2Cl_2, EtOH, MeCN, DMSO using 10.00 mm quartz cells. The excitation wavelength was at the absorption maxima. Atmospheric oxygen contained in solutions was not removed. Concentration of the compounds in the solution was 5.0×10^{-5} M and 5.0×10^{-6} M for absorption and fluorescence measurements, respectively. The relative fluorescence quantum yields (Φ_F) were determined using quinine sulfate (5×10^{-5} M) in 0.1 M H_2SO_4 as a standard ($\Phi_F = 0.546$).

Absolute quantum yield for solid state and time-resolution study were recorded on Horiba FlouroMax 4 Spectrofluorimeter (Kyoto, Japan) with Quanta-φ integrating sphere using FluorEssence 3.5 Software.

Table S2. Photophysical data of acids **1a**, **b** and salts **2a**, **b** at different concentrations in DMSO, 1,4-dioxane, and MeOH[a]

Entry	Compd	Solvent	Concentration, μM	UV-Vis		Fluorescence		Stokes shift, nm/cm^{-1}
				λ_{max}, nm	ε, M·cm^{-1}	λ_{em}, nm	QY, %	
1	**1a**	1,4-dioxane	50	336	11500	414	57.9	78/5607
2			30	336	10700	414	80.8	78/5607
3			10	337	11200	414	81.7	77/5519
4			5	340	10600	414	90.5	74/5257
5			1	340	16000	414	95.3	74/5257
6		MeOH	50	328	13700	419	34.2	91/6621
7			30	325	13800	410	50.8	85/6379
8			10	325	16100	406	68.1	81/6139
9			5	325	14200	406	81.2	81/6139
10			1	325	24000	406	85.4	85/6379
11		DMSO	50	336	12400	426	49.0	90/6288
12			30	336	12600	425	59.7	89/6232
13			10	334	12500	425	71.8	91/6411
14			5	334	12800	425	78.6	91/6411
15			1	331	22000	425	84.7	94/6682
16	**1b**	1,4-dioxane	50	342	11150	421	54.6	79/5487
17			30	342	11200	421	76.5	79/5487
18			10	340	11200	421	77.0	81/5659
19			5	340	11400	421	82.6	81/5659
20			1	340	15000	421	91.3	81/5659
21		MeOH	50	336	13800	433	26.9	97/6667
22			30	333	14100	419	38.1	86/6164
23			10	332	15700	413	42.0	81/5907
24			5	332	17800	413	43.5	81/5907
25			1	332	18000	412	56.8	85/5849
26		DMSO	50	342	11800	441	45.2	99/6564
27			30	340	12000	440	59.3	100/6684
28			10	340	12000	440	72.5	100/6684
29			5	340	11800	440	73.1	100/6684
30			1	340	20000	440	80.5	100/6684
31	**2a**	DMSO	50	328	20000	395	1.2	67/5171
32			30	328	20100	399	3.0	71/5425
33			10	326	20300	400	4.9	74/5675
34			5	321	20000	416	10.6	95/7114
35			1	321	44000	422	17.0	101/7456

36	**2b**	DMSO	50	340	18000	408	0.7	68/4902
37			30	340	18100	408	1.2	68/4902
38			10	340	18800	408	2.0	68/4902
39			5	340	19000	413	3.2	73/5199
40			1	340	33000	435	7.4	95/6423

a - Concentration for absorption measurements, c = 5 × 10^{-5} M; for fluorescence spectra, c = 5 × 10^{-6} M and $\lambda_{ex} = \lambda_{abs.}$

Table S3. Photophysical investigation data for acids **1a**, **b** in different concentrations in mixture of DMSO-H_2O, 1,4-dioxane-H_2O, MeOH-H_2O (v/v, 1/9) and salts **2a**, **b** in H_2O

Entry	Compd	Solvent	Concentration, µM	UV-Vis		Fluorescence		Stokes shift, nm/cm^{-1}
				λ_{max}, nm	ε, M·L^{-1}	λ_{em}, nm	QY, %	
1	**1a**	1,4-dioxane-water	50	322	11400	420	34	98/7246
2			10	322	12500	420	65	99/7343
3			5	322	13000	420	66	98/7246
4			1	316	15000	420	70	104/7836
5		MeOH-water	50	320	15000	421	26	101/7497
6			10	320	15100	420	60	100/7440
7			5	320	15200	420	61	100/7440
8			1	320	25000	420	62	100/7440
9		DMSO-water	50	322	12000	422	30	100/7359
10			10	322	12800	421	60	99/7303
11			5	322	13200	420	64	98/7246
12			1	322	16000	420	88	100/7359
13	**1b**	1,4-dioxane-water	50	328	11600	431	21	103/7286
14			10	328	11900	430	40	102/7232
15			5	328	13000	430	42	102/7232
16			1	323	21000	430	46	107/7704
17		MeOH-water	50	327	13000	431	19	104/7459
18			10	325	11200	429	42	104/7459
19			5	325	12200	429	47	104/7459
20			1	325	19000	429	56	104/7459
21		DMSO-water	50	328	12400	431	20	103/7286
22			10	329	13000	431	39	102/7193
23			5	330	13200	430	44	100/7047
24			1	330	20000	430	51	100/7047
25	**2a**	water	50	320	12300	423	31	103/7609
26			10	320	12300	423	64	103/7609
27			5	320	12600	423	68	103/7609
28			1	320	17000	423	80	103/7609
29	**2b**	water	50	326	13000	432	19	106/7527
30			10	326	12400	432	42	106/7527
31			5	325	12600	432	44	107/7621
32			1	325	17000	432	46	107/7621

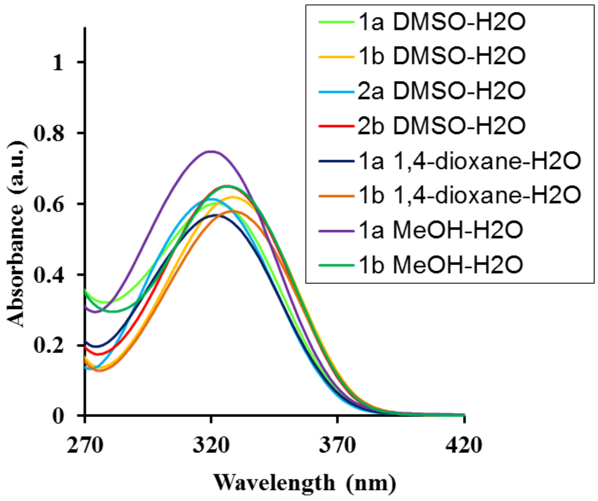

a

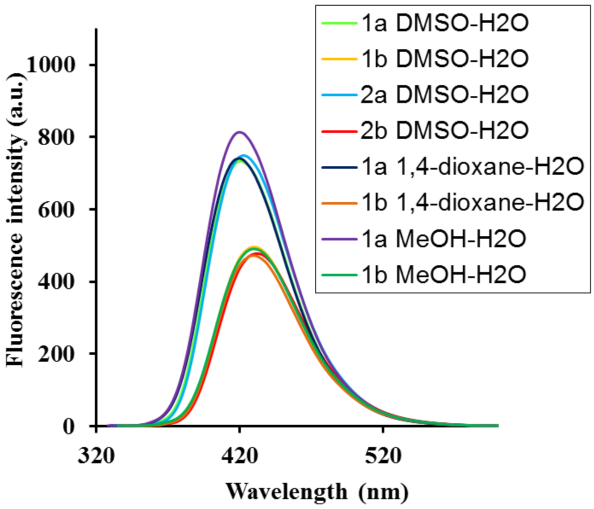

b

Fig. S5. (*a*) Absorption and (*b*) fluorescence spectra of acids **1a, b** and salts **2a, b** in a solvent-H_2O mixture (v/v 1/9). Concentration for absorption measurements, c = 5 × 10^{-5} M; for fluorescence spectra, c = 5 × 10^{-6} M and $\lambda_{ex} = \lambda_{abs}$.

Table S4. Lifetime values of **1a, b** and **2a, b** obtained in DMSO, DMSO-H_2O (D-W, 1/9, v/v), Dioxane, Dioxane-H_2O (Diox-W, 1/9, v/v), MeOH, MeOH-H_2O (MeOH-W, 1/9, v/v), H_2O *c* = 5×10^{-6} M

Entry	Compd	Compd	$\tau_1{}^a$ (ns)	$A_1{}^b$	$\tau_2{}^a$ (ns)	$A_2{}^b$	$\tau_3{}^a$ (ns)	$A_3{}^b$	$\langle\tau\rangle_f{}^c$ (ns)	$\chi^{2\ d}$
1	**1a**	DMSO	3.02	0.21	6.78	0.79			5.99	1.15
2	**1a**	D-W	2.83	0.35	4.50	0.65			3.92	1.02
3	**1b**	DMSO	3.31	0.22	7.67	0.78			6.71	1.06
4	**1b**	D-W	2.58	0.70	4.80	0.30			3.25	1.17
5	**1a**	Dioxane	2.48	0.31	5.74	0.69			4.73	1.19
6	**1a**	Diox-W	1.87	0.15	3.93	0.85			3.62	1.08
7	**1b**	Dioxane	2.61	0.35	6.35	0.64			4.87	1.11
8	**1b**	Diox-W	2.43	0.59	4.01	0.41			3.07	1.06
9	**1a**	MeOH	2.65	0.62	5.09	0.38			3.58	1.00
10	**1a**	MeOH-W	2.69	0.26	4.17	0.74			3.78	1.11
11	**1b**	MeOH	1.93	0.83	5.51	0.17			2.54	1.02
12	**1b**	MeOH-W	2.47	0.62	3.74	0.38			2.95	1.13
13	**2a**	DMSO	1.81	0.10	6.74	0.90			6.25	1.11
14	**2a**	Water	2.51	0.16	4.15	0.84			3.89	1.15
15	**2b**	DMSO	0.28	0.39	1.27	0.08	7.58	0.52	4.15	1.02
16	**2b**	Water	2.34	0.26	3.35	0.74			3.08	1.14

[a]Fluorescence lifetime. [b]Fractional contribution. [c]Weighted mean lifetime. [d]χ^2-Chi-squared.

Table S5. Lifetime values of **1a,b** and **2a,b** obtained in DMSO, DMSO-H_2O (D-W, 1/9, v/v), Dioxane, Dioxane- H_2O (Diox-W, 1/9, v/v), MeOH, MeOH-H_2O (MeOH-W, 1/9, v/v), H_2O c = 1×10^{-6} M

Entry	Compd	Solvent	τ_1[a] (ns)	A_1[b]	τ_2[a] (ns)	A_2[b]	τ_3[a] (ns)	A_3[b]	$\langle\tau\rangle_f$[c] (ns)	χ^2 [d]
1	**1a**	DMSO	2.20	0.22	6.65	0.78			5.67	1.08
2	**1a**	DMSO-W	2.81	0.38	6.22	0.62			4.92	1.19
3	**1b**	DMSO	2.10	0.08	7.62	0.92			7.18	1.10
4	**1b**	D-W	2.70	0.65	3.42	0.35			2.95	1.12
5	**1a**	Dioxane	0.98	0.25	5.73	0.75			4.54	1.01
6	**1a**	Diox-W	1.03	0.25	3.85	0.75			3.15	1.06
7	**1b**	Dioxane	1.08	0.16	6.25	0.84			5.42	1.07
8	**1b**	Diox-W	1.09	0.19	2.96	0.67	4.98	0.14	2.89	1.19
9	**1a**	MeOH	0.61	0.12	2.56	0.58	4.96	0.30	3.05	1.01
10	**1a**	MeOH-W	0.55	0.60	3.94	0.94			4.03	1.12
11	**1b**	MeOH	2.01	0.85	5.36	0.15			2.51	1.05
12	**1b**	MeOH-W	2.78	0.62	3.68	0.38			3.12	1.18
13	**2a**	DMSO	2.04	0.09	6.68	0.91			6.26	1.16
14	**2a**	H_2O	1.95	0.01	3.98	0.99			3.96	1.07
15	**2b**	DMSO	2.94	0.11	7.66	0.78	0.89	0.11	6.40	1.14
16	**2b**	H_2O	2.18	0.07	3.28	0.93			3.20	1.07

[a] Fluorescence lifetime. [b]Fractional contribution. [c]Weighted mean lifetime. [d]χ^2-Chi-squared.

5. NMR spectra of acids 1a and 1b in different solvents

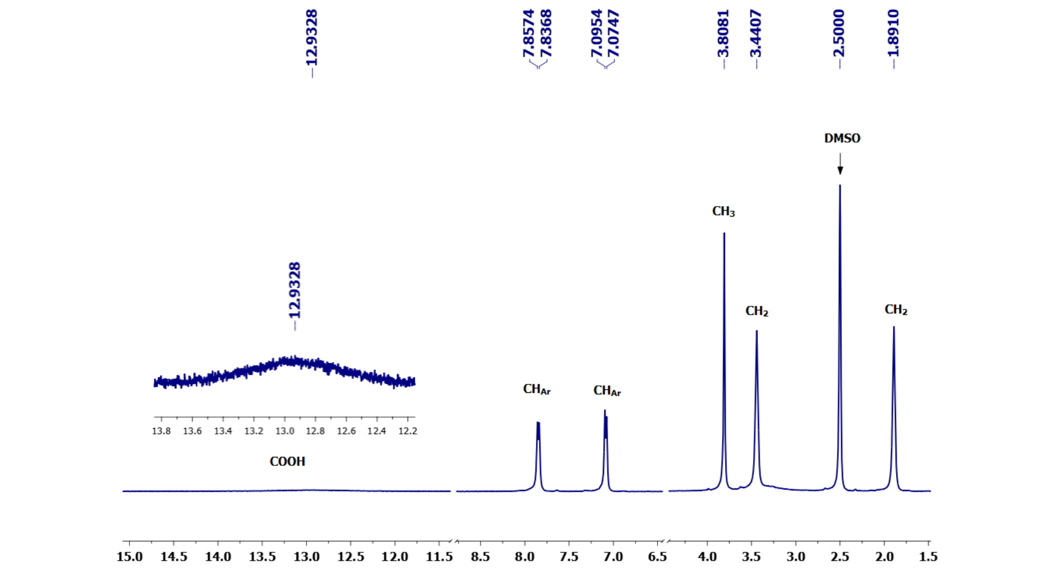

1a (c = 7×10^{-2} M)

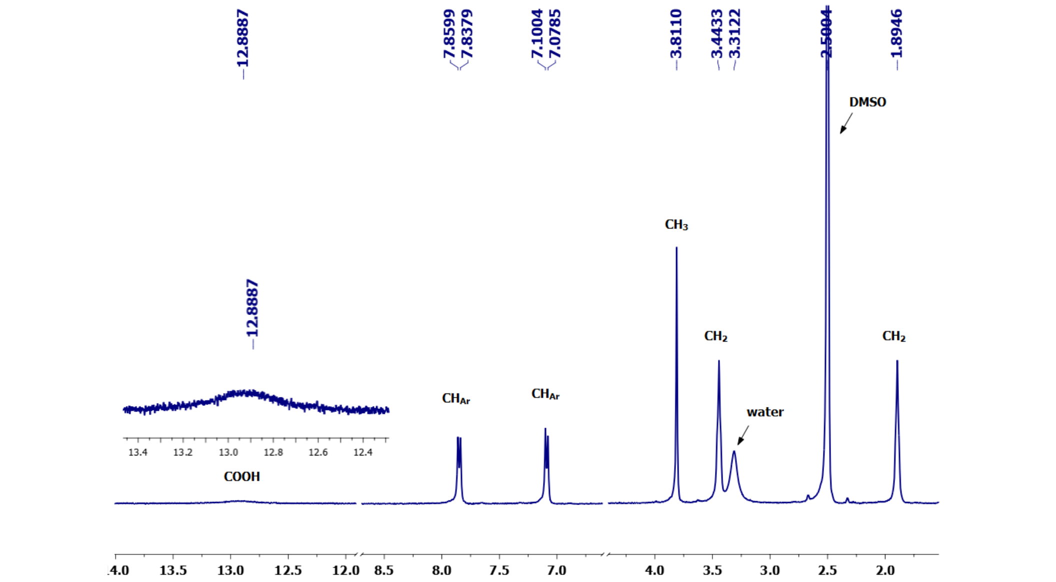

1a (c = 7×10^{-3} M)

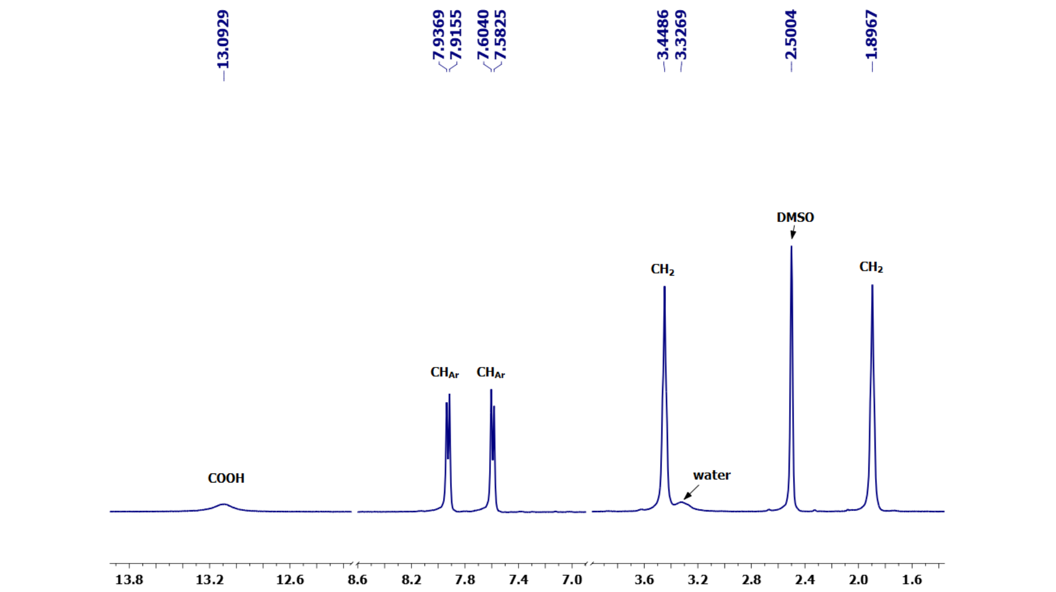

1b (c = 7×10^{-2} M)

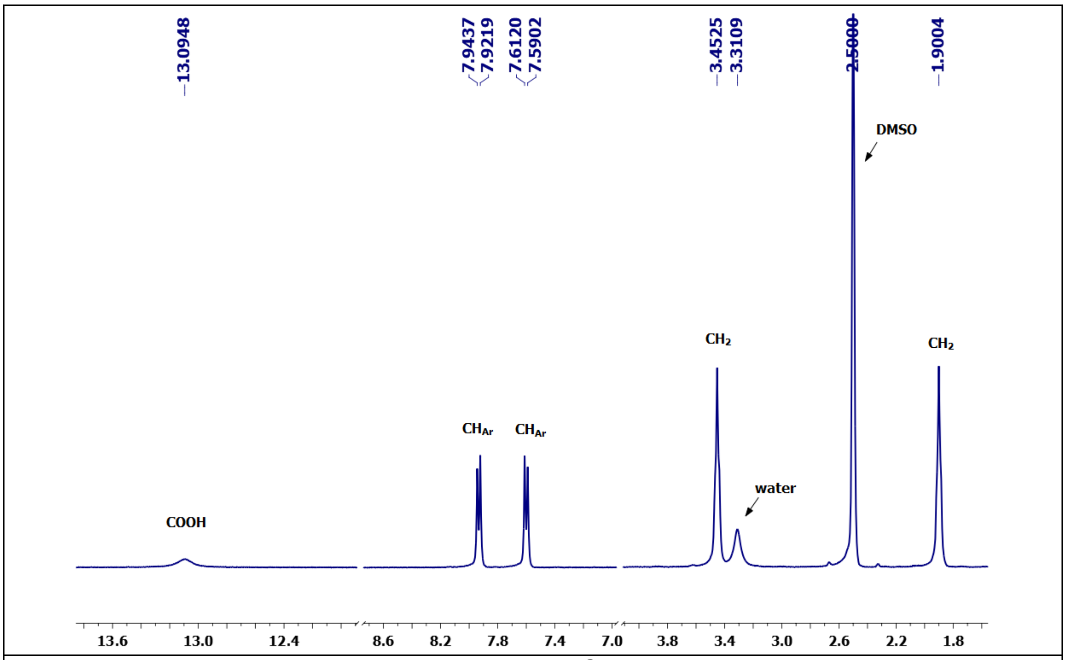

1b (c = 7×10^{-3} M)

Fig. S6. ^{1}H NMR ^{1}H spectra for the acids **1a** and **1b** in DMSO-d$_6$

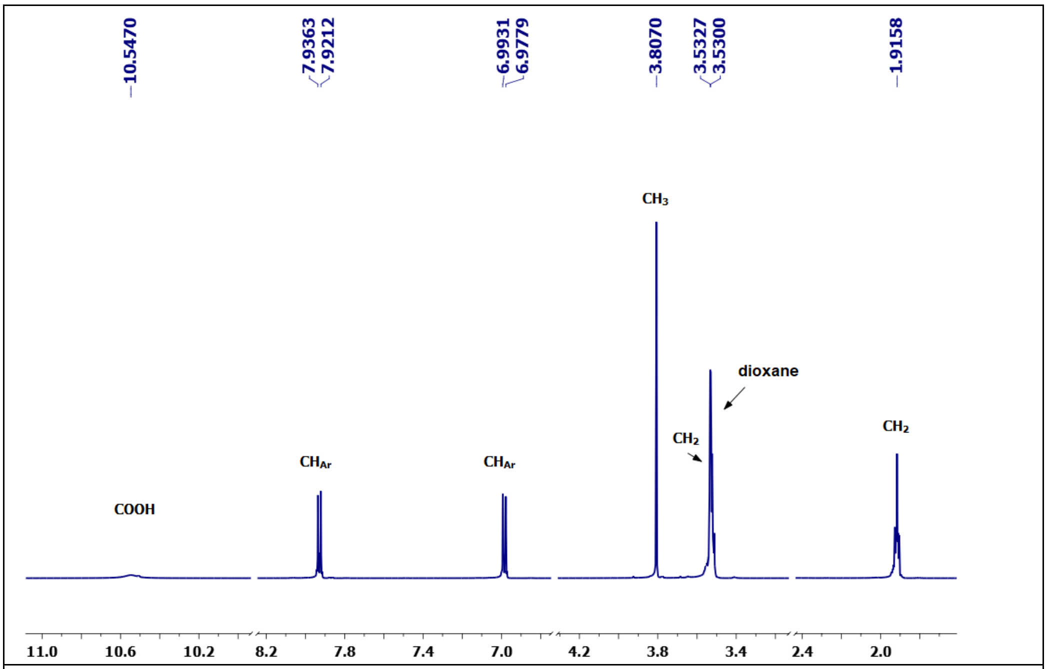

1a (c = 7×10^{-2} M)

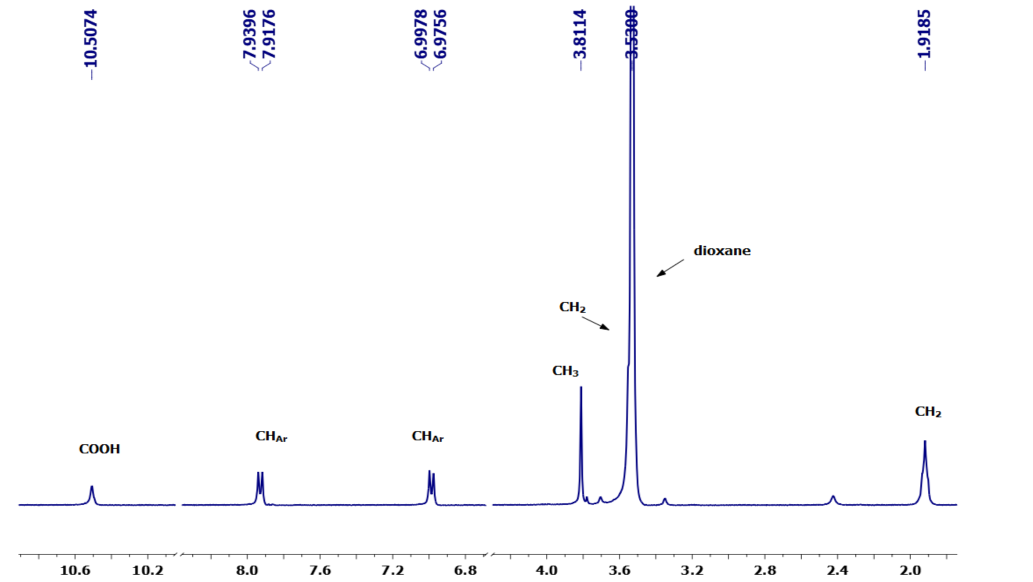

1a ($c = 7\times10^{-3}$ M)

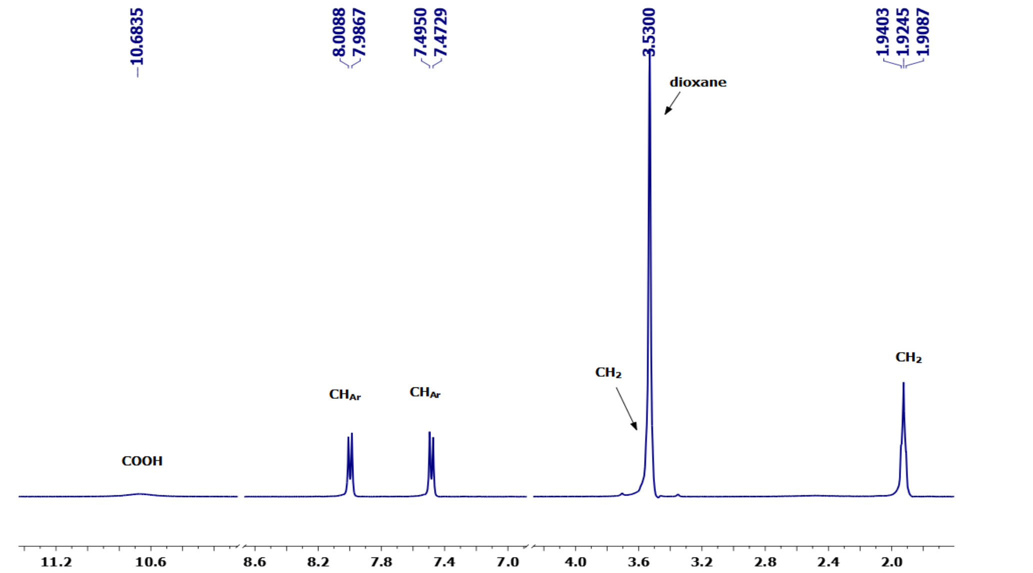

1b ($c = 7\times10^{-2}$ M)

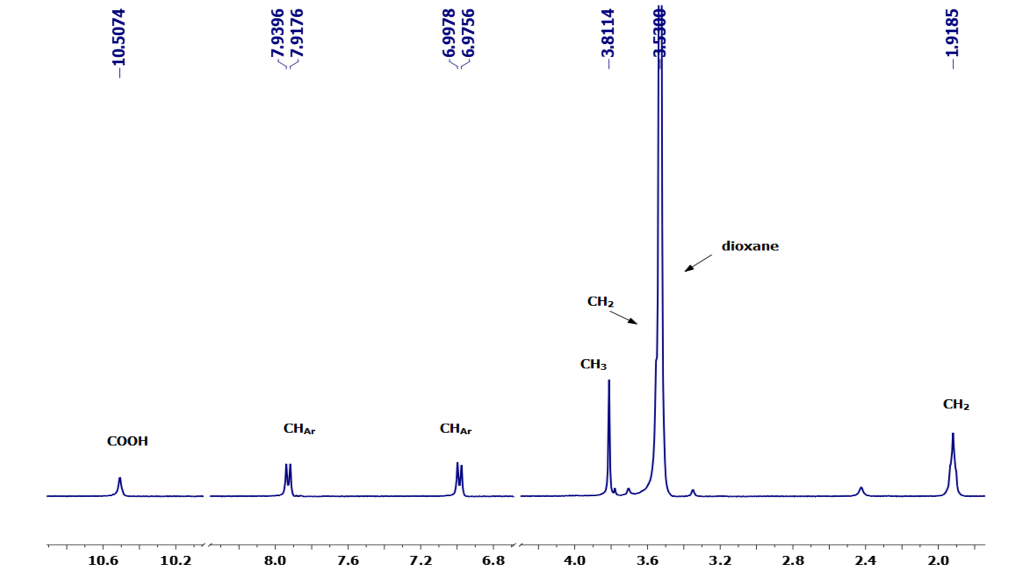

1b ($c = 7\times10^{-3}$ M)

Fig. S7. ^{1}H NMR spectra for the acids **1a** and **1b** in 1,4-dioxane-d_8

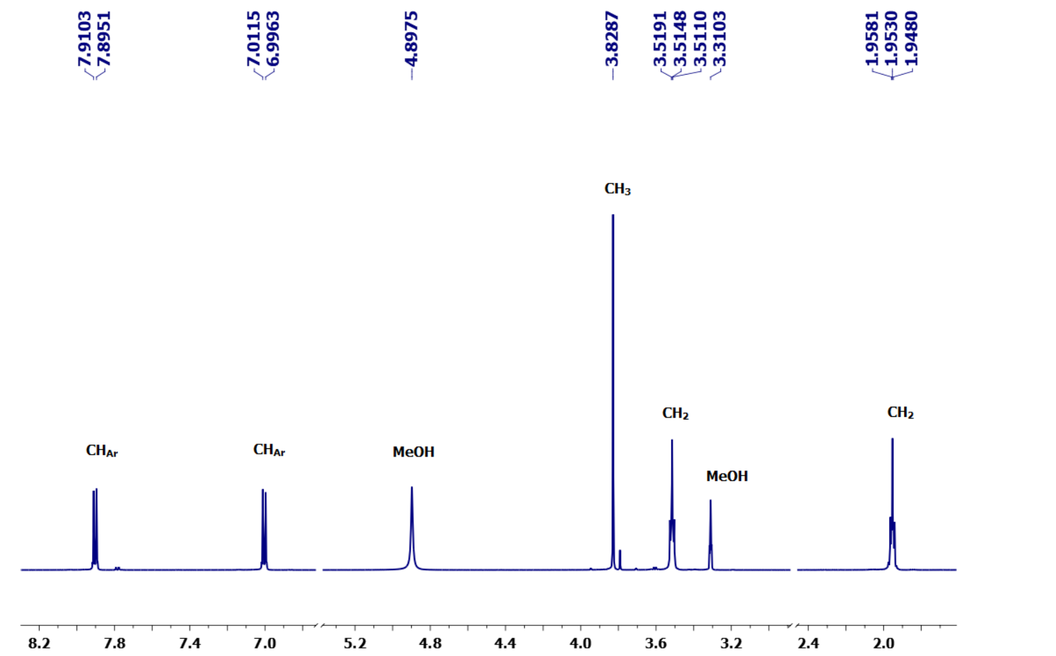

1a (c = 7×10^{-2} M)

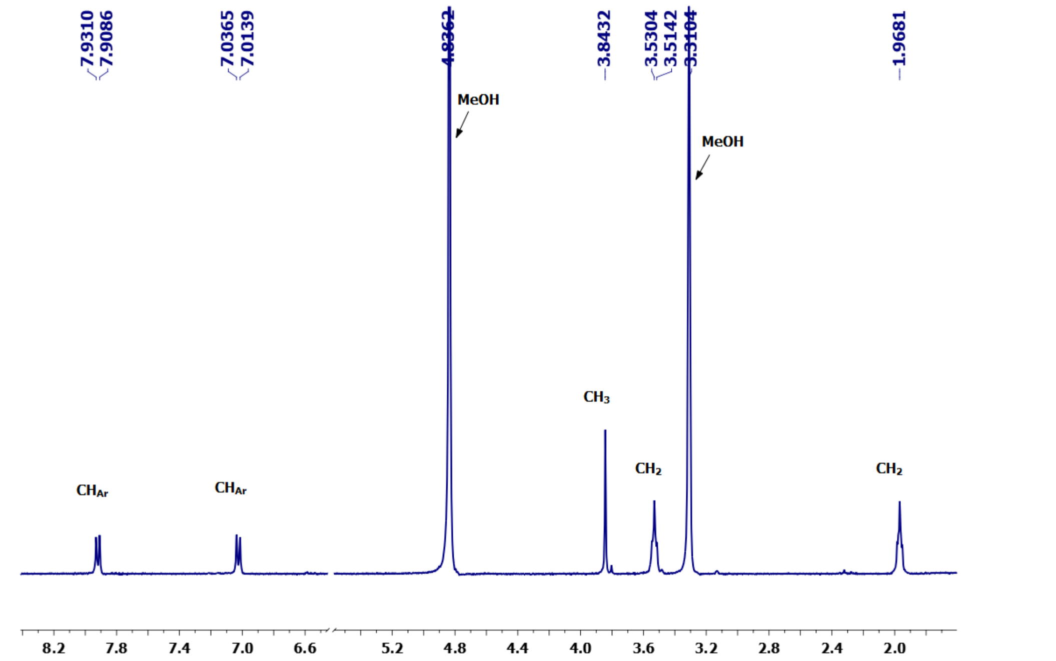

1a (c = 7×10^{-3} M)

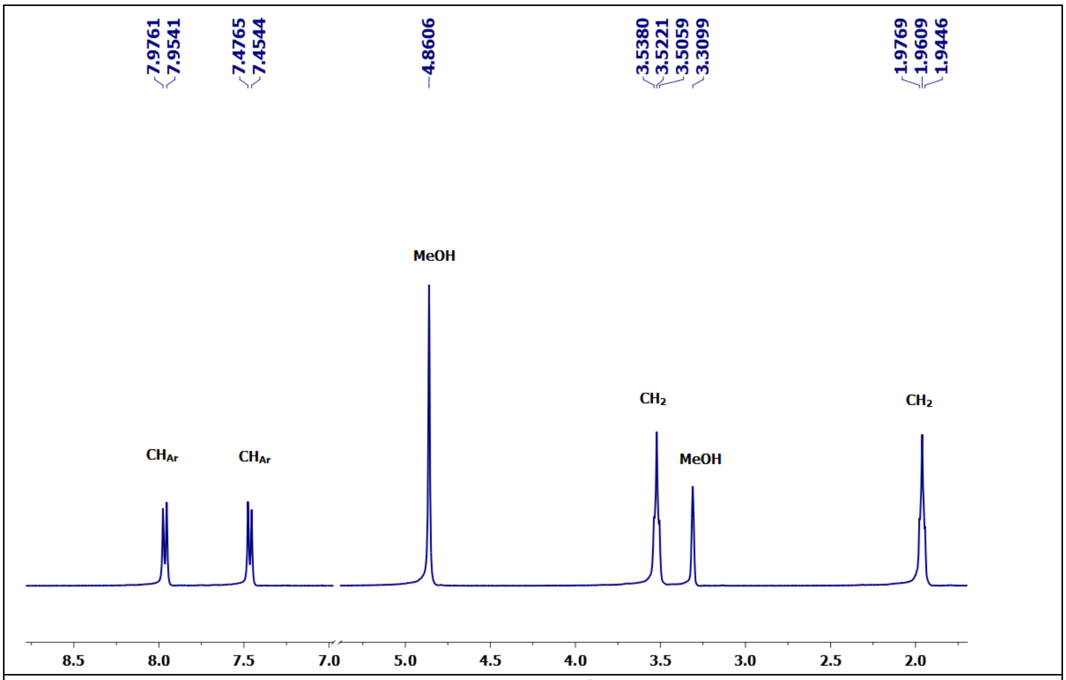

1b (c = 7×10^{-2} M)

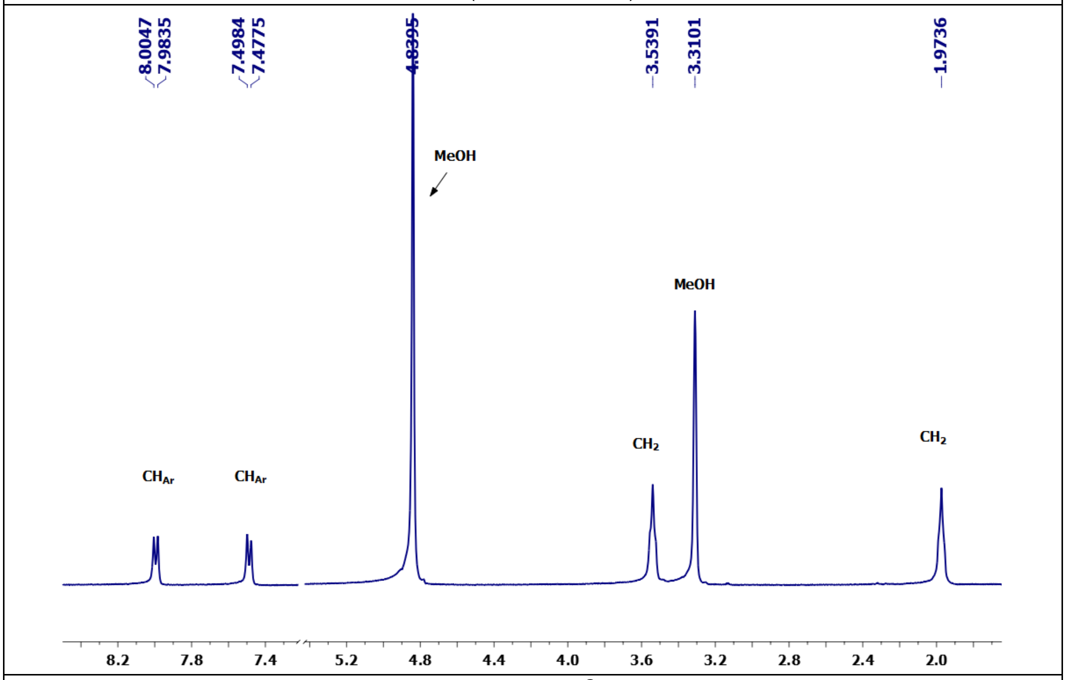

1b (c = 7×10^{-2} M)

Fig. S8. ^{1}H NMR spectra for the acids **1a** and **1b** in MeOH-d_4

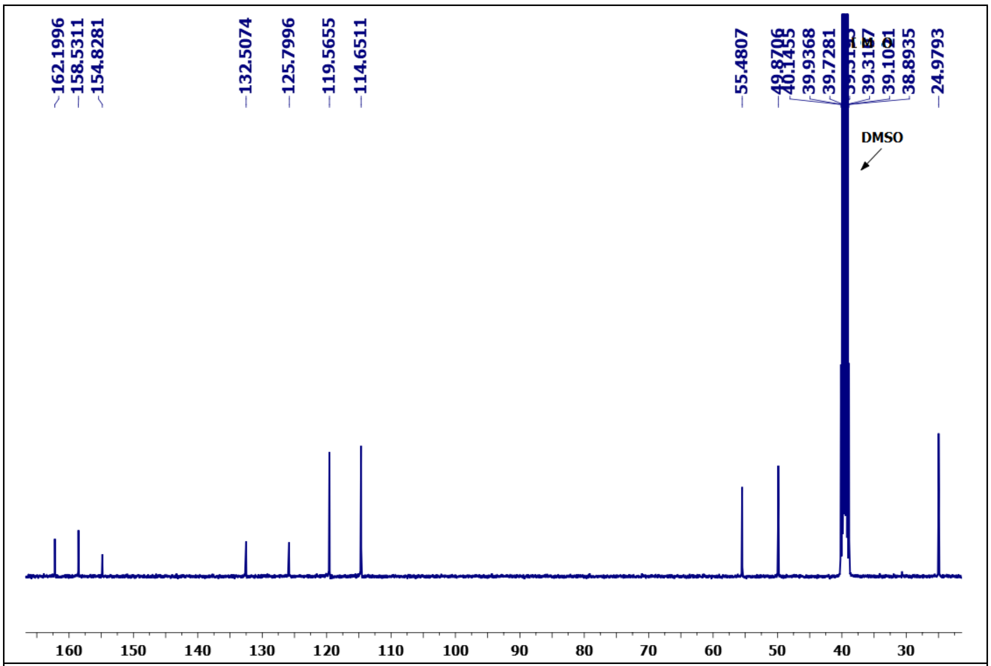

1a ($c = 7\times10^{-2}$ M)

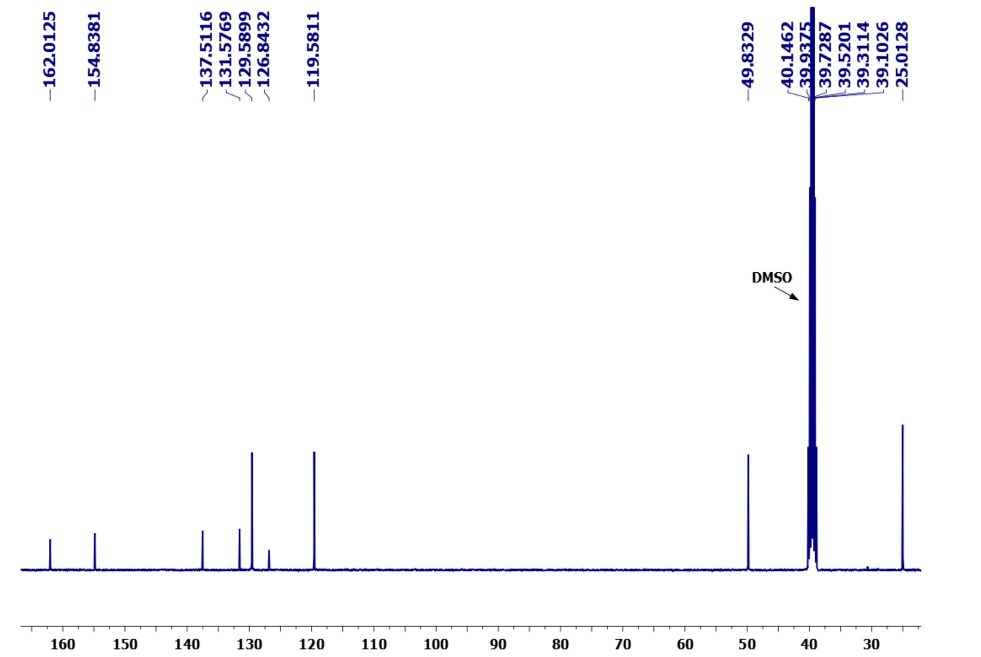

1b ($c = 7\times10^{-2}$ M)

Fig. S9. ^{13}C NMR spectra for the acids **1a** and **1b** in DMSO-d_6

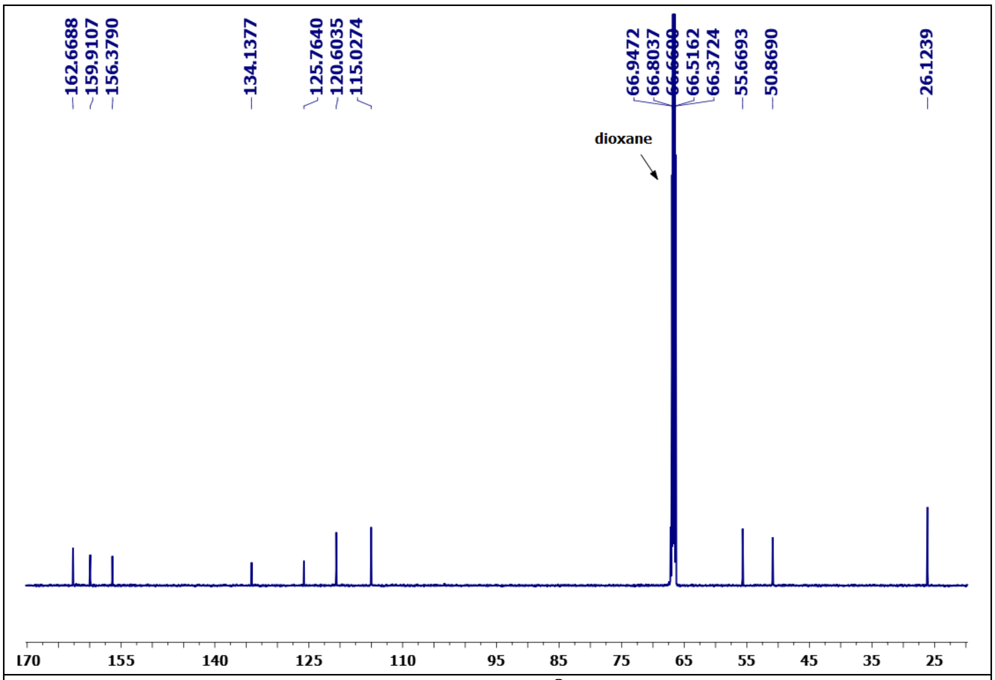

1a (c = 7×10^{-2} M)

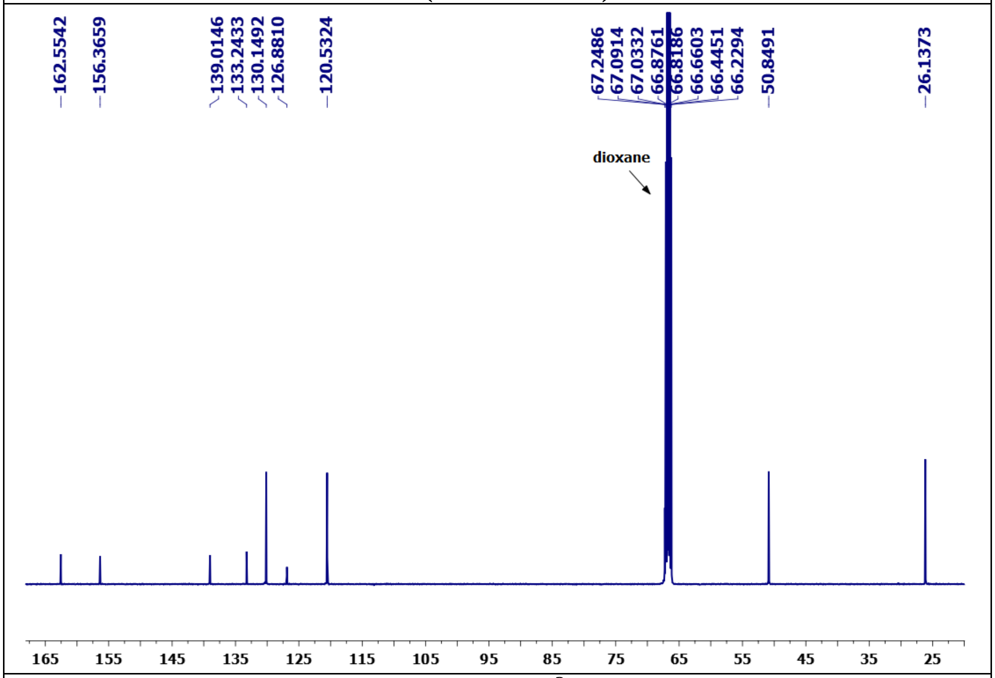

1b (*c* = 7×10^{-2} M)

Fig. S10. ^{13}C NMR spectra for the acids **1a** and **1b** in 1,4-dioxane-d_8

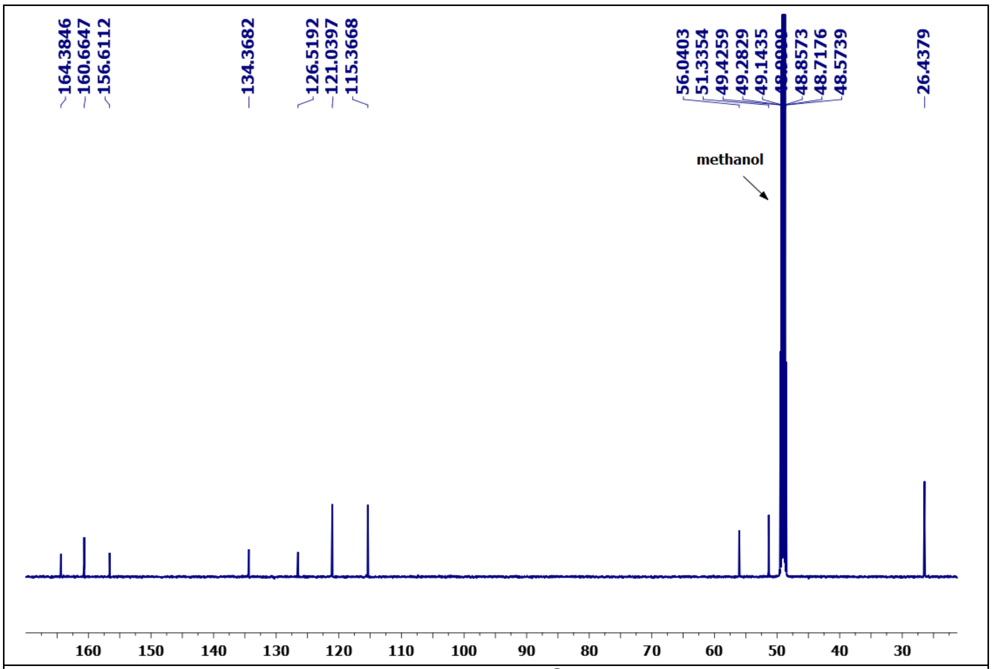

1a (c = 7×10^{-2} M)

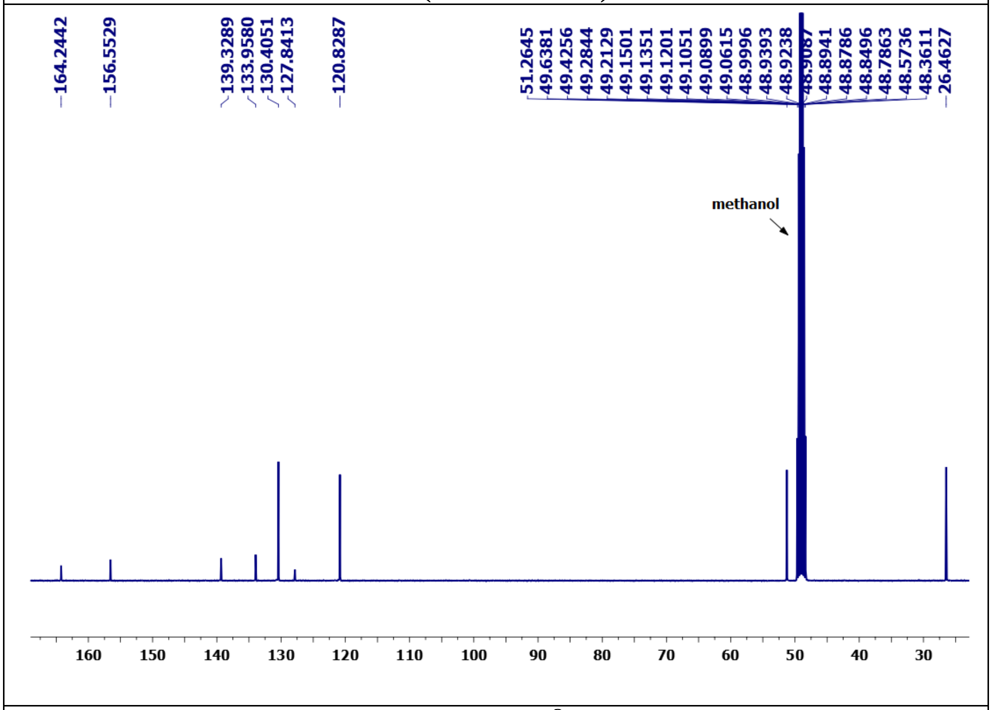

1b (c = 7×10^{-2} M)

Fig. S11. ^{13}C NMR Spectra for the acids **1a** and **1b** in MeOH-d$_4$

Table S6a. Calculated ^{1}H NMR Chemical Shifts in compounds **1a** and **1b** in gas phase and in different solvents.

Compound	solvent	CH_2 H16'	CH_2 H16	CH_2 H17'	CH_2 H17	CH_2 H15'	CH_2 H15	CH_2 H18	CH_2 H18'	OMe H19,H19'	OMe H19''	CH_{Ar} H6	CH_{Ar} H2	CH_{Ar} H3	CH_{Ar} H5	COOH H13
**1a**	Gas	2.19	2.09	2.09	2.09	3.53	3.83	3.01	5.18	3.91	4.17	7.44	7.44	8.54	8.71	6.34
	Methanol	2.19	2.09	2.09	2.09	3.53	3.83	3.01	5.19	3.90	4.17	7.43	7.43	8.53	8.71	6.32
	DMSO	2.19	2.09	2.09	2.09	3.53	3.83	3.01	5.18	3.90	4.17	7.44	7.44	8.53	8.71	6.33
	1,4-dioxane	2.11	2.01	2.11	2.01	3.52	3.82	2.93	5.31	3.82	4.11	7.22	7.39	8.52	8.70	5.97
**1b**	Gas	2.19	2.10	2.10	2.10	3.55	3.84	3.05	5.16	-	-	7.95	7.95	8.58	8.67	6.42
	Methanol	2.19	2.10	2.10	2.10	3.55	3.84	3.05	5.16	-	-	7.95	7.95	8.57	8.67	6.40
	DMSO	2.19	2.10	2.10	2.10	3.55	3.84	3.05	5.16	-	-	7.95	7.95	8.57	8.67	6.41
	1,4-dioxane	2.12	2.00	2.18	2.05	3.53	3.84	2.97	5.29	-	-	7.84	7.84	8.52	8.66	6.07

Table S6b. Scaled ^{1}H NMR Chemical Shifts in compounds **1a** and **1b** in different solvents.

Compound	solvent	CH_2 H16'	CH_2 H16	CH_2 H17'	CH_2 H17	CH_2 H15'	CH_2 H15	OMe H19,H19'	OMe H19''	CH_{Ar} H6	CH_{Ar} H2	CH_{Ar} H3	CH_{Ar} H5
**1a**	Methanol	2.16	2.07	2.07	2.07	3.37	3.65	3.71	3.96	6.92	6.92	7.92	8.08
	DMSO	2.10	2.01	2.01	2.01	3.33	3.61	3.67	3.92	6.93	6.93	7.93	8.1
	1,4-dioxane	2.12	2.03	2.12	2.03	3.41	3.68	3.68	3.95	6.79	6.94	7.98	8.14
**1b**	Methanol	2.16	2.08	2.08	2.08	3.39	3.66	-	-	7.39	7.39	7.95	8.04
	DMSO	2.10	2.02	2.02	2.02	3.35	3.62	-	-	7.40	7.40	7.97	8.06
	1,4-dioxane	2.13	2.02	2.18	2.06	3.42	3.70	-	-	7.36	7.36	7.98	8.11

Table S7a. Calculated ^{13}C NMR Chemical Shifts in compounds **1a** and **1b** in gas phase and in different solvents.

Compound	Solvent	CH_2 C15	CH_2 C18	CH_2 C16	CH_2 C17	OMe C19	C_{Ar} C1	C_{Ar} C2	C_{Ar} C6	C_{Ar} C3	C_{Ar} C5	C_{Ar} C4	C_{Het} C8	C_{Het} C9	COOH C11
**1a**	Gas	46.8	46.5	22.1	24.5	50.9	160.5	116.9	109.2	119.2	118.6	132.5	155.1	121.9	163.3
	Methanol	46.8	46.5	22.1	24.5	50.9	160.5	116.9	109.1	119.3	118.6	132.5	155.1	121.9	163.3
	DMSO	46.8	46.5	22.1	24.5	50.9	160.5	116.9	109.2	119.2	118.6	132.5	155.1	121.9	163.3
	1,4-dioxane	46.6	46.4	22.3	24.6	50.5	160.0	116.8	108.0	119.6	118.6	133.0	154.8	122.0	163.0
**1b**	Gas	47.1	46.5	22.2	24.5	-	155.0	119.1	118.4	129.5	123.4	138.2	139.8	129.5	163.2
	Methanol	47.0	46.5	22.2	24.5	-	155.0	119.0	118.4	129.6	123.4	138.2	139.8	129.5	163.2
	DMSO	47.0	46.5	22.2	24.5	-	155.0	119.0	118.4	129.5	123.4	138.2	139.8	129.5	163.2
	1,4-dioxane	46.8	46.4	22.4	24.6	-	154.7	118.9	118.5	128.9	123.4	138.2	140.0	129.1	162.9

Table S7b. Scaled ^{13}C NMR Chemical Shifts in compounds **1a** and **1b** in different solvents.

Compound	Solvent	CH_2 C15	CH_2 C18	CH_2 C16	CH_2 C17	OMe C19	C_{Ar} C1	C_{Ar} C2	C_{Ar} C6	C_{Ar} C3	C_{Ar} C5	C_{Ar} C4	C_{Het} C8	C_{Het} C9	COOH C11
**1a**	Methanol	50.76	50.47	26.62	28.97	54.77	161.87	119.26	111.64	121.61	120.92	134.51	156.59	124.15	164.60
	DMSO	49.63	49.34	25.60	27.93	53.62	160.23	117.82	110.33	120.06	119.47	133.00	154.98	122.68	162.96
	1,4-dioxane	50.30	50.10	26.63	28.87	54.09	160.75	118.67	110.10	121.40	120.42	134.45	155.68	123.74	163.67
**1b**	Methanol	50.96	50.47	26.72	28.97	-	141.64	131.58	131.67	120.73	121.31	140.08	156.49	125.61	164.51
	DMSO	49.82	49.34	25.70	27.93	-	140.10	130.08	130.17	119.28	119.86	138.54	154.88	124.14	162.86
	1,4-dioxane	50.49	50.10	26.72	28.87	-	141.27	130.65	130.46	120.33	120.72	139.51	155.58	125.10	163.57

Table S8. Calculated NBO charges in the hydrogen atoms of compound **1a** in gas phase and in different solvents.

solvent	CH_2 H16'	CH_2 H16	CH_2 H17'	CH_2 H17	CH_2 H15'	CH_2 H15	CH_2 H18'	CH_2 H18	OMe H19,H19'	OMe H19''	CH_{Ar} H6	CH_{Ar} H2	CH_{Ar} H3	CH_{Ar} H5	COOH H13
Methanol	0.219	0.207	0.205	0.219	0.201	0.207	0.236	0.185	0.182	0.201	0.235	0.234	0.245	0.244	0.506
DMSO	0.219	0.207	0.205	0.219	0.201	0.207	0.236	0.186	0.183	0.201	0.235	0.234	0.245	0.244	0.506
1,4-dioxane	0.215	0.204	0.205	0.216	0.200	0.206	0.241	0.181	0.178	0.198	0.227	0.230	0.243	0.243	0.497
gas	0.219	0.207	0.205	0.219	0.201	0.207	0.235	0.186	0.183	0.201	0.236	0.234	0.245	0.244	0.507

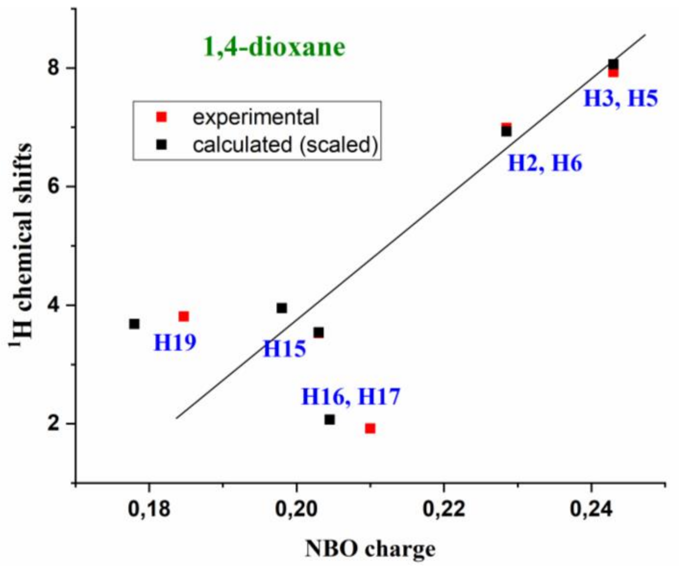

Fig. S12. Relationships between the theoretically scaled and experimental ^{1}H chemical shifts in 1,4-dioxane versus the theoretical NBO atomic charges.

5. References

1. Gavlik, K. D.; Lesogorova, S. G.; Sukhorukova, E. S.; Subbotina, J. O.; Slepukhin, P. A.; Benassi, E. 2016, 2700-2710.

2. Safronov, N. E.; Fomin, T. O.; Minin, A. S.; Todorov, L.; Kostova, I.; Benassi, E.; Belskaya, N. P. Dyes and Pigm. 2020, 178, 108343.

3. Reijenga, J.; van Hoof, A.; van Loon, A.; Teunissen, B. Development of Methods for the Determination of pKa Values. Anal. Chem. Insights 2013, 8, 53−71.
